# Scanning of Genetic Variants and Genetic Mapping of Phenotypic Traits in Gilthead Sea Bream Through ddRAD Sequencing

**DOI:** 10.3389/fgene.2019.00675

**Published:** 2019-08-06

**Authors:** Dimitrios Kyriakis, Alexandros Kanterakis, Tereza Manousaki, Alexandros Tsakogiannis, Michalis Tsagris, Ioannis Tsamardinos, Leonidas Papaharisis, Dimitris Chatziplis, George Potamias, Costas S. Tsigenopoulos

**Affiliations:** ^1^School of Medicine, University of Crete, Heraklion, Greece; ^2^Foundation for Research and Technology–Hellas (FORTH), Heraklion, Greece; ^3^Institute of Marine Biology, Biotechnology and Aquaculture (IMBBC), Hellenic Center for Marine Research (HCMR) Crete, Greece; ^4^Deparment of Economics, University of Crete, Gallos Campus, Rethymnon, Greece; ^5^Department of Computer Science, University of Crete, Voutes Campus, Heraklion, Greece; ^6^Nireus Aquaculture SA, Koropi, Greece; ^7^Department of Agriculture Technology, Alexander Technological Education Institute of Thessaloniki, Thessaloniki, Greece

**Keywords:** aquaculture, *Sparus aurata*, double digest random amplified DNA, Genome Wide Association Study, feature selection

## Abstract

Gilthead sea bream (Sparus aurata) is a teleost of considerable economic importance in Southern European aquaculture. The aquaculture industry shows a growing interest in the application of genetic methods that can locate phenotype–genotype associations with high economic impact. Through selective breeding, the aquaculture industry can exploit this information to maximize the financial yield. Here, we present a Genome Wide Association Study (GWAS) of 112 samples belonging to seven different sea bream families collected from a Greek commercial aquaculture company. Through double digest Random Amplified DNA (ddRAD) Sequencing, we generated a per-sample genetic profile consisting of 2,258 high-quality Single Nucleotide Polymorphisms (SNPs). These profiles were tested for association with four phenotypes of major financial importance: Fat, Weight, Tag Weight, and the Length to Width ratio. We applied two methods of association analysis. The first is the typical single-SNP to phenotype test, and the second is a feature selection (FS) method through two novel algorithms that are employed for the first time in aquaculture genomics and produce groups with multiple SNPs associated to a phenotype. In total, we identified 9 single SNPs and 6 groups of SNPs associated with weight-related phenotypes (Weight and Tag Weight), 2 groups associated with Fat, and 16 groups associated with the Length to Width ratio. Six identified loci (Chr4:23265532, Chr6:12617755, Chr:8:11613979, Chr13:1098152, Chr15:3260819, and Chr22:14483563) were present in genes associated with growth in other teleosts or even mammals, such as semaphorin-3A and neurotrophin-3. These loci are strong candidates for future studies that will help us unveil the genetic mechanisms underlying growth and improve the sea bream aquaculture productivity by providing genomic anchors for selection programs.

## Introduction

The gilthead sea bream, Sparus aurata (Linnaeus, 1758), is a teleost fish of great economic importance for the Mediterranean aquaculture industry (Tsigenopoulos et al., 2014). It ranks first among other aquacultured species in South Mediterranean with total production of 160,563 tons for 2016 (FEAP, 2017). One of the top interests of the aquaculture industry is the genetic improvement of the stocks to maximize the efficiency of the production and the product quality (Fernandes et al., 2017). Coupled with this concern, various areas of sea bream biology are being explored, such as nutrition requirements (Silva-Merrero et al., 2017; Guardiola et al., 2018), immune responses (Antonopoulou et al., 2017; Bahi et al., 2018; Tapia-Paniagua et al., 2018), skeletal development (Negrín Báez et al., 2015; Vélez et al., 2018), reproduction, and broodstock management (Loukovitis et al., 2011). Recently, the genome of sea bream has been sequenced and analysed offering a backbone for conducting genomic analyses on the species (Pauletto et al., 2018).

One of the main avenues to genetically improve the cultured stock is to identify associations between genetic variants and traits of interest, such as growth, disease resistance, and fat content. Genome Wide Association Studies (GWAS) offer the way to accomplish this by comparing the genotypes of individuals having varying phenotypes for a specific trait of interest. GWAS have boosted the field of human genetics as well as plant and livestock breeding (Geng et al., 2017), leading to improved higher selection accuracies of the animal breeding programmes, which in turn leads to lower costs and greater yield (Geng et al., 2017). To conduct a GWAS experiment in non-model species, genome-wide sampling of genetic variants is required. Application of double digest Random Amplified DNA (ddRAD) leads to thousands of polymorphic loci that require sophisticated strategies for data analysis (Catchen, 2013) and is widely used for GWAS studies (Baird et al., 2008; Etter et al., 2011; Anderson et al., 2012; Palaiokostas et al., 2013). It is well known that biological datasets are susceptible to the curse of dimensionality (Lie, 2014; Stephens et al., 2015). Various methods have been developed to solve such complicated problems, such as feature selection (Tsagris et al., 2018a). Feature selection (FS) is used to identify the important, predictive genetic variants by removing the noise propagated by redundant features, i.e., markers that have the same genotypic profile across all samples. Several FS algorithms have been developed like (Fontanarosa and Dai, 2011), Orthogonal Matching Pursuit (OMP) (Cai and Wang, 2011), and Statistically Equivalent Signature (SES) (Lagani et al., 2017), differing mainly in the approach to discover associations and the computational efficiency.

In aquaculture breeding programs, these features-markers can be used for marker assisted selection (MAS) (Yue, 2014). However, genome-wide variants can also be used to directly evaluate breeders, the so-called genomic selection (GS) method (Yue, 2014). Genomic selection is a breeding value estimation methodology that aims to increase the rate of genetic gain, leading to improvement of certain phenotypes *via* genetic marker utilization (Heffner et al., 2011; Lorenz et al., 2011; Yue, 2014; Khatkar, 2017). Genetic markers associated with production traits are used to predict breeding values with high accuracy (Goddard and Hayes, 2007; Sonesson and Meuwissen, 2009; Wang et al., 2017, Gutierrez et al., 2015). Although high availability of genetic markers (i.e., SNP markers) could be used for the improvement of the accuracy of breeding value estimation through the use of a Genomic Relationship matrix (i.e., GBLUP), some genetic markers that are also associated with production traits could further increase the accuracy of breeding value estimation and, moreover, allow for the inclusion of alternative models of inheritance, rather than only additive, in the genetic evaluation procedures. Genomic selection based on specific traits such as fat, weight, and disease resistance can have great effects on the productivity and profitability of several aquaculture species (Yue, 2014).

In this study, we sought to identify genetic markers associated with important phenotypes in sea bream. We used ddRAD sequencing to identify and genotype genome-wide single nucleotide polymorphisms (SNPs) in multiple sea bream families. We performed both GWAS and FS to test the association among a combination of loci and the phenotypes of fat, weight, tag weight, and length/width. Finally, genomic prediction of the phenotypes was tested using the selected polymorphisms to evaluate its potential in selection for improved phenotypic traits like weight in sea bream. Our ultimate goal was to construct a signature—a combination of genetic markers—that will lead to maximizing the sea bream aquaculture efficiency, by improving the selected phenotypic traits.

## Materials and Methods

### Sample Collection

The fish used in this study were a subset of a larger experiment with progeny from 66 male and 35 female brooders constituting 73 different full sib families from the breeding program of a commercial aquaculture company (Nireus Aquaculture S.A.). From those 73 full sib families, 14 families originating from 13 males and 11 females were selected (selective genotyping), based on their within-family variation of bodyweight at harvest, for genotyping with microsatellite markers in order to perform a QTL confirmation experiment (Chatziplis et al. 2018, in preparation). Seven male and six female brooders with 105 progeny in total, constituting six full sib families and one maternal half sib family (10 progeny on average per family), were used for ddRAD library preparation and sequencing. These seven families were those exhibiting the greatest family variation of bodyweight at harvest out of 14 total families included in the QTL verification experiment (Chatziplis et al. 2018, in preparation). All progeny were reared in commercial conditions, and after PIT tagging, they were transferred to sea cages at 220 Days Post Hatching (DPH) for the growth period. For all progeny, the weight at tagging (g) (205 DPH), weight at harvest (g) (750 DPH), percentage (%) of fat at harvest (as measured in terms of body electrical conductivity, 692 Distell) as described by Besson et al. (2019), the total length at harvest (cm) (750 DPH), and the width at harvest (cm) (750 DPH) were measured.

### Library Preparation and Sequencing

Individual DNA library preparation and sequencing of the samples, which were extracted using a modified salt-based extraction protocol based on Miller et al. (1988) and treated with RNase to remove residual RNA, were performed. Genomic DNA was eluted in 5 mmol/L Tris, pH 8.5, and stored in 4°C. Each sample was quantified by spectrophotometry (Nanodrop 1000–Thermo Fisher Scientific) and quality assessed by 0.7% agarose gel electrophoresis. To build the ddRAD library, we used the protocol described by Manousaki et al. (2016), with some minor modifications. Briefly, each of 144 DNA samples (13 parents in triplicates and 105 offspring; 21 ng DNA per sample) was separately but simultaneously digested by two high-fidelity restriction enzymes (RE): SbfI (CCTGCA|GG recognition site) and SphI (GCATG|C recognition site), both sourced from New England Biolabs (NEB), UK. Digestions were incubated at 37°C for 90 min, using 10 U of each enzyme per microgram DNA in 1 CutSmart Buffer (NEB), in a 6 µl total reaction volume. The reactions were slowly cooled to room temperature, and 3 µl of a premade adapter mix was added to the digested DNA and incubated at room temperature for 10 min. This adapter mix contained individual-specific combinations of P1 (SbfI-compatible) and P2 (SphI-compatible) adapters at 6 and 72 nM concentrations, respectively, in 1· reaction buffer 2 (NEB). The ratio of P1 to P2 adapter (1:12) was selected to reflect the relative abundance of SbfI and SphI cut sites present. P1 and P2 adapter included an inline five- or seven-base barcode for sample identification. Ligations were implemented over 3 h at 22°C by addition of a further 3 µl of a ligation mix comprising 4 mM rATP (Promega, UK) and 2000 cohesive-end units of T4 ligase (NEB) in 1· CutSmart buffer (NEB). The ligated samples were pooled together, and the single pool was column-purified (MinElute PCR PurificationKit, Qiagen, UK) and eluted in 70 µl EB buffer (Qiagen, UK). The size selection was performed by agarose gel separation, keeping the fragments between 400 and 700 bp. Following gel purification (MinElute Gel Extraction Kit, Qiagen, UK), the eluted size-selected template DNA (68 µl in EB buffer) was PCR amplified (15 cycles PCR; 32 separate 12.5 µl reactions, each with 1 µl template DNA) using a high-fidelity Taq polymerase (Q5 Hot Start High-Fidelity DNA Polymerase, NEB). The PCR reactions were combined (400 µl total) and column-purified (MinElute PCR Purification Kit). The 57 µl eluate, in EB buffer, was then subjected to a further size-selection clean-up using an equal volume of AMPure magnetic beads (Perkin-Elmer, UK) to maximize removal of small fragments. The final libraries were eluted in 24 µl EB buffer. Lastly, the ddRAD libraries were sequenced in one HiSeq 2500 lane (2x125 bp reads).

### Raw Read Quality Control and Demultiplexing

We used FastQC v.0.11.5 software to check the quality control of the raw sequence data (Andrews and Babraham Bioinformatics Group, 2010). To recover the reads belonging to each individual, we then cleaned and demultiplexed the raw data using Process radtags program from STACKS v.1.46 software (Catchen, 2013). In this step, -c parameter was used to remove reads with an uncalled base, -q parameter was used to discard sequencing reads of low quality (below 20) using the Phred scores provided from the FASTQ files (Catchen, 2013), and -t parameter was set to 100 to truncate final reads length to 100 bp.

### Data Alignment Against Sea Bream Reference Genome

The annotated reference genome of gilthead sea bream has been provided by Hellenic Centre for Marine Research (H.C.M.R.) (Accession Numbers: SRR6244977-SRR6244982) (Pauletto et al., 2018). To align our samples to the reference genome, we used Bowtie2 v.2.3.0 (Langmead and Salzberg, 2012) with the following parameters: {end-to-end {sensitive {no-unal. Then, we removed multi-aligned reads, reads with >3 mismatches, and reads with mapping quality lower than 20 with Samtools (Li et al., 2009).

### Genotyping RAD Alleles

Genotypes of each sample were constructed using STACKS pipeline (Catchen, 2013). For each individual, pstacks program was used to build the rad loci based on the alignment on the reference genome, setting the minimum depth of coverage to create a stack (-m) equal to 3 (default) (Paris et al., 2017). Then, a catalogue of loci was constructed using only the parental reads on cstacks program, using default parameters. To match the data of each offspring separately against the respective catalogue, we used sstacks program with ––aligned parameter. Finally, to retrieve the vcf file with the genotypes, we used populations program.

### Kinship

To check family relationship and indicate possible pedigree errors, we used KING v.2.1 software (Manichaikul et al., 2010). Kinship coefficients have been estimated by KING, setting the ––degree parameter equal to 10. Kinship coefficient is a measurement of kinship between two individuals; 1 means homozygous twins, 0 means unrelated (Manichaikul et al., 2010). Finally, to see the genetic distances of studied individuals, we performed a Principal Components analysis (PCA) and Hierarchical clustering, using Euclidean distance. Both PCA and Hierarchical clustering were implemented in R using prcomp and hclust functions, respectively.

### Linear Mixed Models

To fit the mixed model for every phenotype, we used the command lmer from the lme4 R package (Bates et al., 2014). Random effects were fitted for each family to control for the correlation within the families. In mathematical notation, the linear mixed model is written as

(1) $$y_{i}=a+\tau_{i}+\overset{p}{\underset{j=1}{\sum }}\beta_{j}X_{j}+e_{i}$$

where i = 1,…,K, with K denoting the number of families and *y_i_* is the vector of measurements of the i-th family containing n_i_ measurements with $\sum_{\left(i=1\right)}^{K}n_{i}=n$, the overall sample size. The term τ_i_ is the overall constant term. The *τ_i_* is the random effect of the i-th family, the deviation of the i-th family from the overall constant a. The term *β_j_* is the fixed regression coefficient of the variable *X_j_*, and *e_i_* is the vector of residuals of the i-th family. The model has two sources of variation: one stemming from the residuals and one stemming from the repeated measurements, $e_{ij}\;N\left(0,\sigma_{e}^{2}\right)$ and $\tau_{i}\;N\left(0,\sigma_{\tau }^{2}\right)$, respectively. Residuals represent elements of variation unexplained by the fitted model. Since this is a form of error, the same general assumptions apply to the group of residuals that we typically use for errors in general: one expects them to be normal and approximately independently distributed with a mean of 0 with some constant variance (Bates et al., 2014). To compare two linear mixed models, we used the Bayesian information criterion (BIC). BIC is a criterion for model selection among a finite set of models; the model with the lowest BIC is preferred. It is based on the log-likelihood function and takes into account the number of estimated parameters. When fitting models, it is possible to increase the likelihood by adding parameters, but doing so may result in over-fitting (Vrieze, 2012). BIC attempts to resolve this problem by introducing a penalty term for the number of parameters in the model.

### Genome Wide Association Study

A typical GWAS analysis tests for variant significance in a set of independent samples. The most common source of sample dependence is family relationships. Yet, our study is based on a family designed cohort. For this reason, we applied a family-based test for variant significance. To perform this, we used lmer in order to create a linear mixed model for each phenotype. This model includes family id as a random effect. To correct for multiple testing, we set the significance threshold to 10^–4^, which is the typical significance level a = 0.05 divided to the number of independent SNPs (497) based on linkage disequilibrium (LD) (Johnson et al., 2010; Clarke et al., 2011). We used the plink tool v.1.90 in order to calculate the LD score (––indep-pairwise 50 5 0.05) (Purcell et al., 2007). Finally, we presented the distribution of the p-values across the genome in Manhattan plots, and we tested for possible p-value inflation through Quantile–quantile (QQ) plots. For these plots, we used the GWASTools (Gogarten et al., 2012) library in R (scripts available upon request).

### Feature Selection

The typical GWAS pipeline reveals individual SNPs that are associated with a specific phenotype. One limitation of this pipeline is that it cannot produce signatures that contain combinations of variants. This problem is commonly referred as SNP to SNP interaction induction (Balliu and Zaitlen, 2016). The large number of tested genotypes in a typical GWAS experiment makes prohibitive the efficient computation of variant combinations. Also, the burden of multiple testing increases linearly to the number of combined variants. This means that a SNP–SNP interaction should be of extreme significance in order to be detected by a method that tests all possible combinations of variants. To tackle this problem, we employed a different approach. We considered SNPs as variables that describe a certain phenotype. We then applied methods that seek the optimum subset of variables with which we can construct a predictive model for a trait of interest (e.g., Weight). This approach is called Variable selection, or Feature Selection (FS). Solving the FS problem has numerous advantages (Tsamardinos and Aliferis, 2003). Features in biology (e.g., SNPs and gene expressions) are commonly found to be expensive to measure, store, and process (Stephens et al., 2015). By reducing the number of measurable markers-loci via FS, one can reduce this cost. A high-quality FS algorithm improves the predictive performance of the resulting model by removing the noise propagated by redundant features. For our study, we used two different FS algorithms: The first is the statistically equivalent signature (SES) algorithm, and the second is the Orthogonal Matching Pursuit (OMP) algorithm.

#### The Statistically Equivalent Signature Algorithm

Commonly FS algorithms aim to find a single group of features that has the highest predictive power. On the contrary, SES algorithm introduced by Lagani et al. (2017) attempts to identify multiple signatures (feature subsets) whose performances are statistically equivalent. SES produces several signatures of the same size and predictive power regardless of the limited sample size or high collinearity of the data (Statnikov and Aliferis, 2010). It performs multiple hypothesis tests for each feature, conditioning on subsets of the selected features. For each feature, the maximum p-value of these tests is retained and the feature with the minimum p-value is selected. This heuristic has been proved to control the False Discovery Rate (Tsamardinos and Brown, 2008). SES is specially engineered for small sample sizes and eliminates the need for Bonferroni correction and/or FDR filtering (Lagani et al., 2017). Here, we used an adaptation of the SES algorithm that accommodates repeated measurements (Tsagris et al., 2018a). SES algorithm is influenced by the principles of constraint-based learning of Bayesian networks (Lagani et al., 2017). Bayesian networks are directed acyclic graphs that represent the dependency relationships between variables in a dataset. An edge A → B in a Bayesian graph represents the conditional dependence of variable B from variable A. There is a theoretical connection between S and the Bayesian (causal) network that describes best the data at hand (Tsamardinos and Aliferis, 2003). Following the Bayesian networks terminology, the Markov Blanket (MB) of a variable or node A in a Bayesian network is the set of nodes ∂A composed of A’s parents (direct causes), its children (direct effects), and its children’s other parents (other direct causes of the A’s direct effects). Every set of nodes in the network is conditionally independent of A when conditioned on the Markov blanket of the node A (∂A as described in formula 2). Thus, the Markov blanket of a node contains the only knowledge needed to predict the behavior of that node.

(2) Pr⁡(A|∂A,B)=Pr⁡(A|∂A)

#### Orthogonal Matching Pursuit Algorithm

Orthogonal Matching Pursuit is an iterative algorithm. At each iteration, it selects the column-marker of the SNP data matrix, which has the greatest correlation with the current residuals (Cai and Wang, 2011). OMP updates the residuals by projecting the observation onto the linear subspace spanned by the columns that have already been selected and then proceeds to the next iteration. No column is selected twice because the residuals are orthogonal to all the selected columns. The algorithm stops when a criterion is satisfied. We have used its generalized form, gOMP, whose stopping criterion is based upon the difference of the BIC score between two successive models. If the difference is lower than a predefined threshold, the algorithm stops. The major advantage of OMP compared with other alternative methods is its simplicity and fast implementation (Cai and Wang, 2011).

### Model Selection Through Cross Validation

The selection of the appropriate algorithm for each dataset is a challenging task. Commonly, a k-fold cross-validation (CV) is used in order to end up with the algorithm with the best fit in the examined dataset. Cross-validation is a model validation technique for assessing the results of a model. It is commonly used for estimating how precisely a predictive model performs in unknown data samples. The standard method of a prediction problem, where a dataset of known data is given, is to split data samples in folds and every time use the n-1 folds as training dataset and the one fold that is left, as test dataset (“unknown data”). The goal of cross validation is to estimate the expected level of fit of a model to a data set that is independent of the data that were used to train the model. This approach limits problems like over-fitting and gives an insight on how the model will generalize to an independent dataset (Tibshirani and Tibshirani, 2009). To compare the algorithms and select the best model (including algorithm and parameters), we performed cross validation by using all but one sample as training set and the remaining sample as test set iterating over all samples, the so-called Leave-One-Out cross validation method.

The different models were assessed based on the sum or errors when assuming that the “unknown data” belong to each family (Equation 3). The model with the lowest mean sum of errors is selected as best model (Equation 4).

(3) $$ErrOB=\overset{m}{\underset{i=1}{\sum }}E{\left(y_{i\left(n_{i}+1\right)}-x_{i\left(n_{i}+1\right)}^{T}\hat{\beta }-z_{i\left(n_{i}+1\right)}^{T}{\hat{b}}_{i}\right)}^{2}/m,$$

where *y_i_*(*n_i_* + 1), *x_i_*(*n_i_* + 1) and *z_i_* (*n_i_* + 1) are, respectively, the outcome and predictors of the new observation in cluster i, and $\hat{\beta }$ and ${\hat{b}}_{i}$ are, respectively, the estimates of *β* and *b_i_* based on all the training data. This can be estimated by the leave-one-out cross validation,

(4) $$LOOCV=\overset{m}{\underset{i=1}{\sum }}\overset{n_{i}}{\underset{j=1}{\sum }}{\left(y_{ij}-x_{ij}^{T}{\hat{\beta }}^{\left[i,j\right]}-z_{ij}^{T}{\hat{b}}_{i}^{\left[i,j\right]}\right)}^{2}/N,$$

where ${\hat{\beta }}^{\left[i,j\right]}$ and ${\hat{b}}_{i}^{\left[i,j\right]}$ are, respectively, the estimates of *β* and *b_i_* based on the training data without subject j in cluster i (Fang 2011).

### Selected SNP Annotation

To identify potential genes that might be affected by the retrieved SNPs, we searched the reference genome and classified the SNPs to those falling within a genic region (located within or in a window of 10Kb upstream or downstream of an annotated gene) and those that do not. If these regions were described as conserved at the genome browser of Gilthead sea bream (http://biocluster.her.hcmr.gr/myGenomeBrowser?search=1&portalname=Saurata_v1) in any of the following species: Stickleback, Asian sea bass, Medaka, Asian swamp eel and Amazon molly, they were considered as conserved.

## Results

### Genotyping RAD Alleles

Illumina sequencing yielded 559,191,588 raw reads. Following quality control, we filtered out ∼ 15.2% due to ambiguous barcodes, ∼ 2.9% due to low quality, and 1% due to the lack of restriction sites. The rest were successfully assigned to individuals (**Supplementary Table 1** with number of reads per individual). After the demultiplexing, the high-quality reads of each sample were aligned against the reference genome. In total, 93% of the reads were mapped. Downstream filtering resulted in further discarding of multi-aligned reads (∼ 8%) and those with more than three mismatches (∼ 2.96%), keeping finally 351,781,485 reads for analysis. This resulted in an average coverage of 188.25. Although we did not experiment with greater values of m and used the default value proposed by STACKS, the sequencing effort was enough to have 188.25 coverage on average (s.e +/− 9.68) for the loci in our study. However it has been suggested that moderate values of m (3–6) (Paris et al., 2017) might not have any effect on the mean coverage of the reconstructed loci on a teleost species. The ddRAD catalogue built from all parental samples consisted of 15,233 SNPs. The used ddRAD protocol has been applied in other sparids as well (Manousaki et al., 2016; Manousaki et al. unpublished data). In all cases, the number of produced SNPs was in the range of 5,000–10,000 per individual (Manousaki et al., 2016; Palaiokostas et al., 2018). In this study and in accordance to this protocol, the SNP catalogue was built using solely parental data. Thus, the discovered SNPs are within the expected range given the following ddRAD protocol. Variants with allele frequency lower than 0.05 (n = 2,065) were filtered out. From the remaining 13,168, we filtered out the SNPs with call rate lower than 90% (n = 7,882). From the remaining 5,286 SNPs, 3,028 had at least one missing value and 2,258 had no missing values.

### Kinship Assignment

To verify the family identity of the studied individual, we used three different methods: King kinship, Principal Component Analysis (PCA), and Hierarchical clustering (**Supplementary Figure 1**). All three resulted in similar results, and they confirmed the tagging family id, except for two samples, one placed in different family (sample 133 that was identified as a member of Family 2 instead of Family 3) and one that was not placed in any family (sample 882). These two samples were discarded and not included in downstream analyses.

### Association Analysis Through GWAS

**Figure 1:**
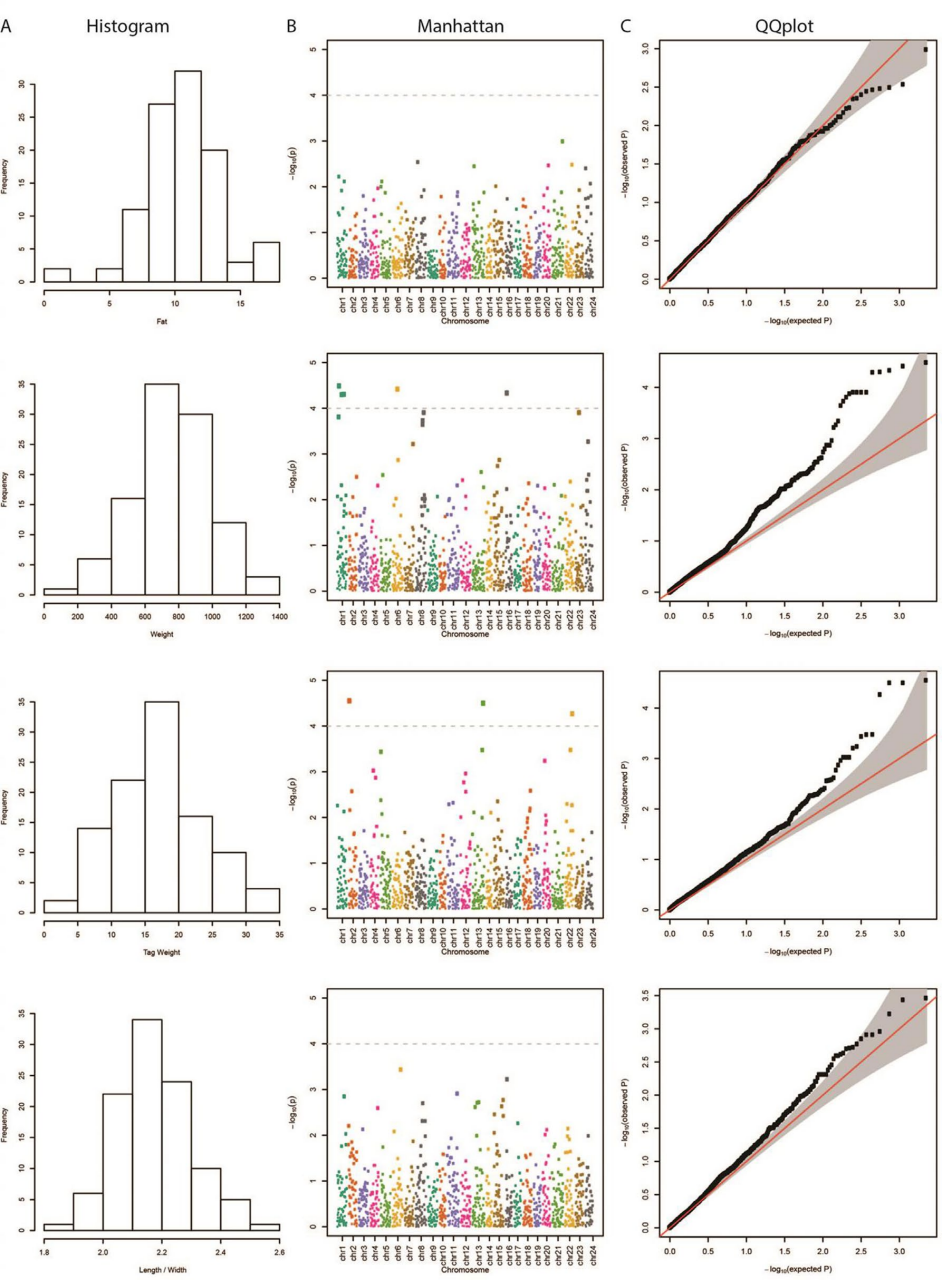
**(A)** Distribution of each examined trait in our samples. **(B)** Manhattan plot demonstrating the locations across the chromosomes of the sea bream genome (horizontal axis) versus the –log (p-values) of the association between the genetic variants and phenotype (vertical axis). The higher the dots, the stronger the genetic association. The significance threshold was set to 10^–4^, in order to correct for multiple testing (dashed line). The different colors represent the different chromosomes. **(C)** Quantile–quantile (QQ) plot of the data shown in the Manhattan plot. The grey area represents the 95% simultaneous confidence bands. Red line is the diagonal (Y = X) or else how the observed data should be placed if they were normally distributed.

The results from the GWAS test among all SNPs and the four phenotypes are shown in **Table 1**. In total, we found five SNPs associated with Weight, four SNPs with Tag Weight, and none for Fat and Length/Width. In **Figure 1**, we show the phenotype distribution, Manhattan plot, and QQ-plot for each phenotype. For illustration purposes, the Manhattan plot depicted was built with variants of known ordered positions on the reference genome. The Manhattan plot for the variants in scaffolds that we do not know the exact position in the genome is given in the **Supplementary Figure 2**. The QQ-plot of Weight revealed a systemic inflation of the observed p-values possibly attributed to the fact that families were selected in such a way as to maximize the weight variation within the cohort. Regarding the loci associated with weight and tag weight, we identified nine SNPs in total (**Table 1**). Five SNPs associated with weight at harvest have been retrieved from the typical GWAS analysis. The first was found in chromosome 1 (chr1:16636968) on “ethanolamine phosphate cytidylyltransferase-like” gene and the second (chr6:12617755) in a conserved region upstream of “myosin-7-like” gene. The third (chr16:2232897) was located on two overlapping genes acetylserotonin O-methyltransferase-like and LBH-like isoform X1. Another two SNPs were found in chromosome 1. The first (chr1.6970078) located downstream of “lymphoid enhancer-binding factor 1” and the second (chr1:20827142) located upstream of “mucin-5AC-like isoform X1” (**Table 1**). Finally, four SNPs (in chromosomes 2, 13, and 22) were associated with weight at tagging. Two were found at “RNA-binding 27 isoform X1” gene (chr13:20975921,chr13:20975924), the third upstream from “Tetratricopeptide repeat 36” gene (Chr2:2623351), and the fourth upstream from “tectonin beta-propeller repeat-containing 2” gene (chr22:18343985).

**Table 1:** Selected SNPs from GWAs analysis using linear mixed models, with significance threshold equal to 10^–^^4^.

Position	Gene	P-value	Beta coefficient	Conserved	Position
Weight					
Chr1:6970078	Lymphoid enhancer-binding factor 1 isoform X1	3.265E−5	174.721	–	Downstream
Chr1:16636968	Ethanolamine-phosphate cytidylyltransferase-like	5.059E−5	189.556	✓	3’UTR
Chr1:20827142	Mucin-5AC-like isoform X1	4.976E−5	−161.835	✓	Upstream
Chr16:2232897	Acetylserotonin O-methyltransferase-like, LBH-like isoform X1	4.648E−5	−338.149	✓	3’UTR
Chr6:12617755	Transmembrane 199	3.838E−5	205.210	✓	Upstream
	myosin-7 like				Downstream
Tag Weight					
Chr13:20975921	RNA-binding 27 isoform X1	3.168E−5	4.748	–	Intron
Chr13:20975924	RNA-binding 27 isoform X1	3.168E−5	4.748	–	Intron
Chr2:2623351	Tetratricopeptide repeat 36	2.823E−5	6.183	–	Upstream
Chr22:18343985	tectonin beta-propeller repeat-containing 2	5.405E−5	−5.139	–	Upstream

### Association Analysis Through FS

**Figure 2:**
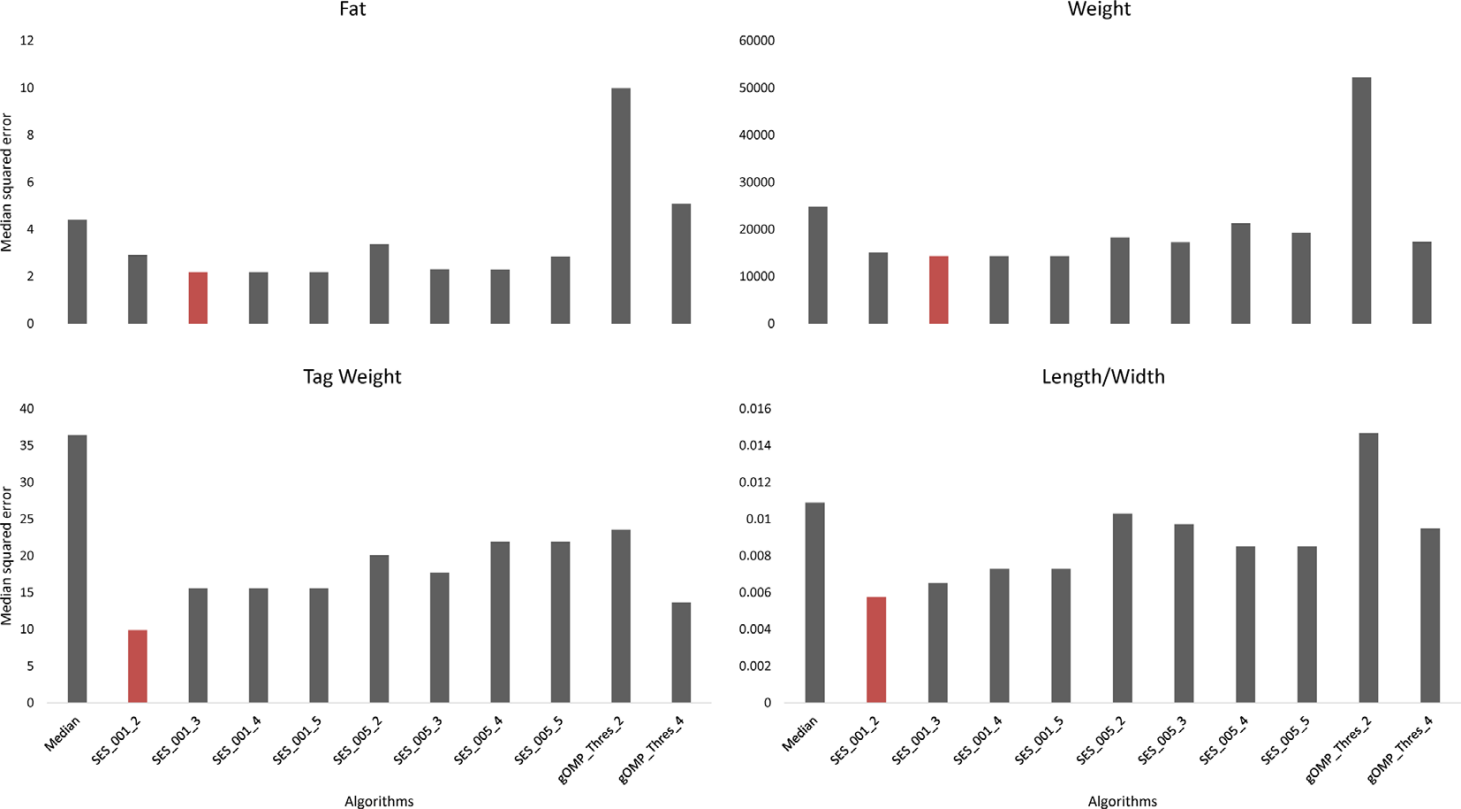
Comparison of different algorithms predicting the traits of interest, based on median squared error, after leave one out cross validation. SES algorithm tested for different thresholds (Threshold equal to 0.01 or 0.05) and for different numbers of SNPs as condition set (k = 2, 3, 4, 5). OMP algorithm tested for different thresholds as stop criterion (Threshold = 2 or 4 units in BIC score).

**Figure 3:**
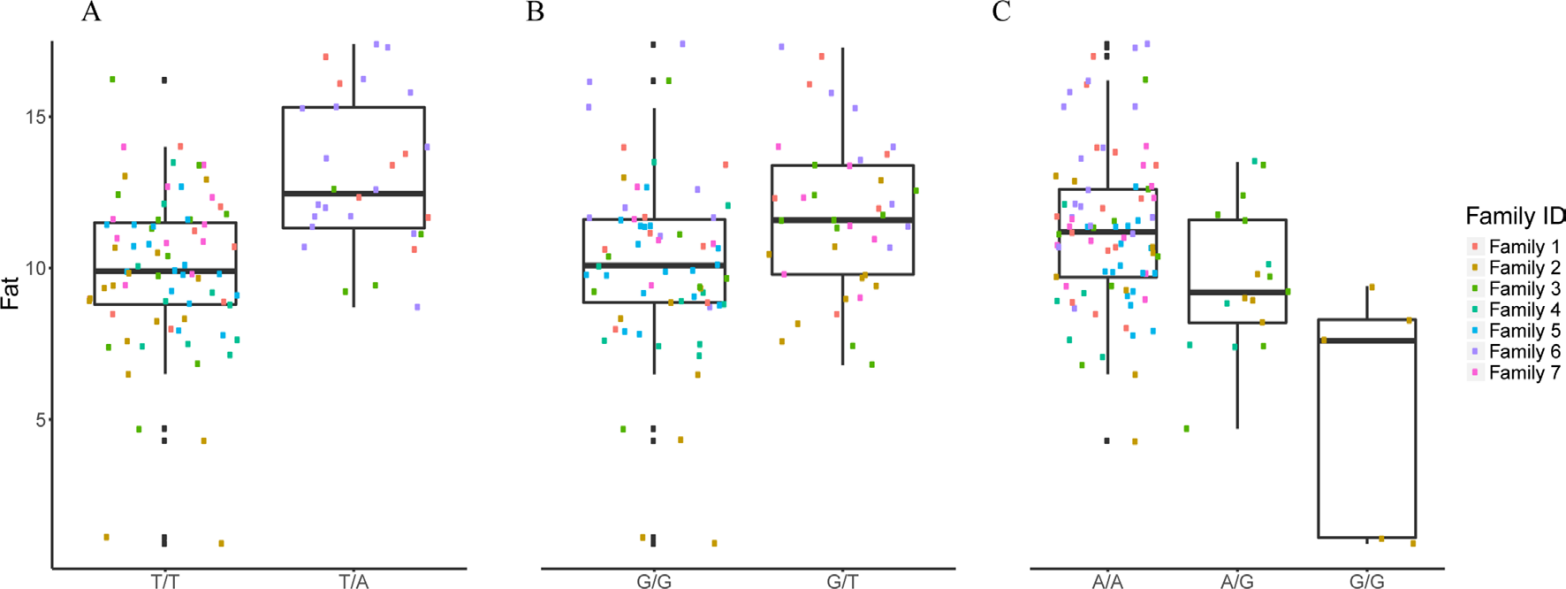
The effect of each of the selected SES SNPs associated with fat content. (A-C) Boxplots of selected SNPs. **(A)** chr8:1385781, **(B)** chr13:1098152, **(C)** chr21:19924408.

**Figure 4:**
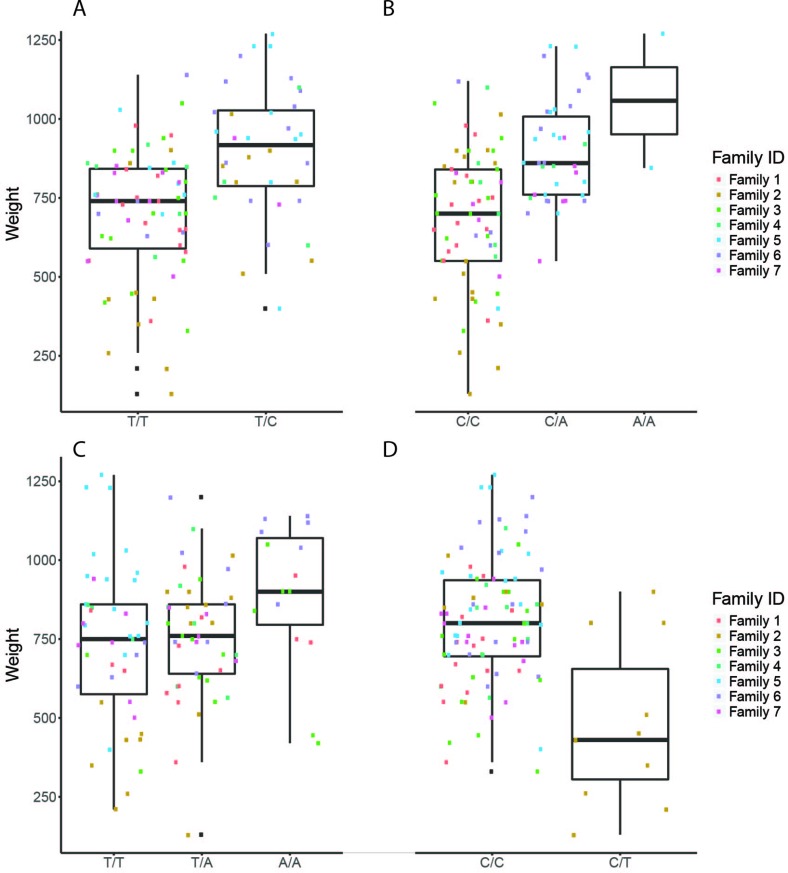
The effect of each of the selected SES SNPs associated with weight at harvest. (A-D) Boxplots of selected SNPs. **(A)** chr1:16636968, **(B)** chr6:12617755, **(C)** chr8:11613979, **(D)** chr16:2232897

**Figure 5:**
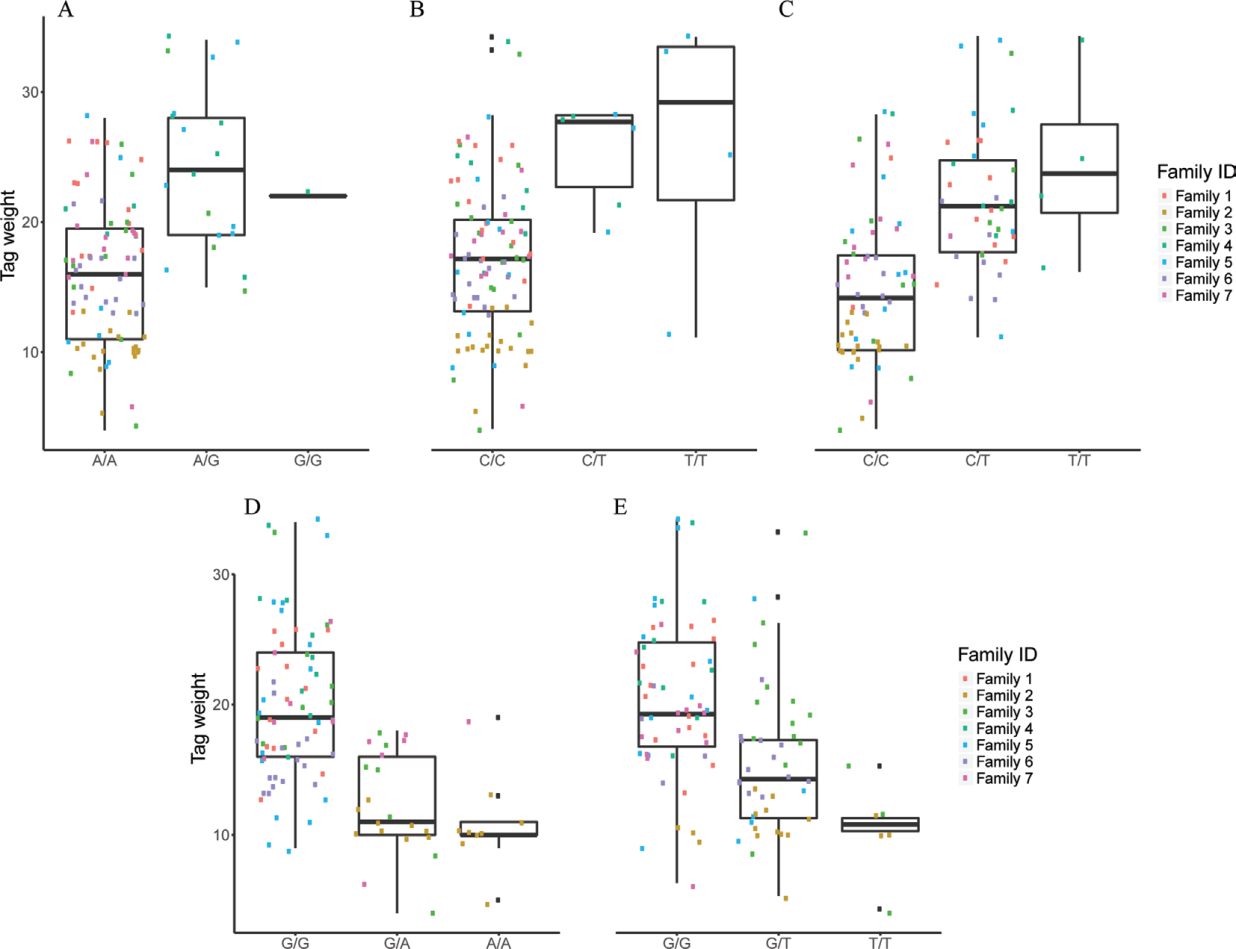
The effect of each of the selected SES SNPs associated with tag weight. (A-E) Boxplots of selected SNPs. **(A)** chr2:2623351, **(B)** chr13:20883924, **(C)** chr13:20975921, **(D)** chr22:18343985, **(E)** scaffold4139:36071.

**Figure 6:**
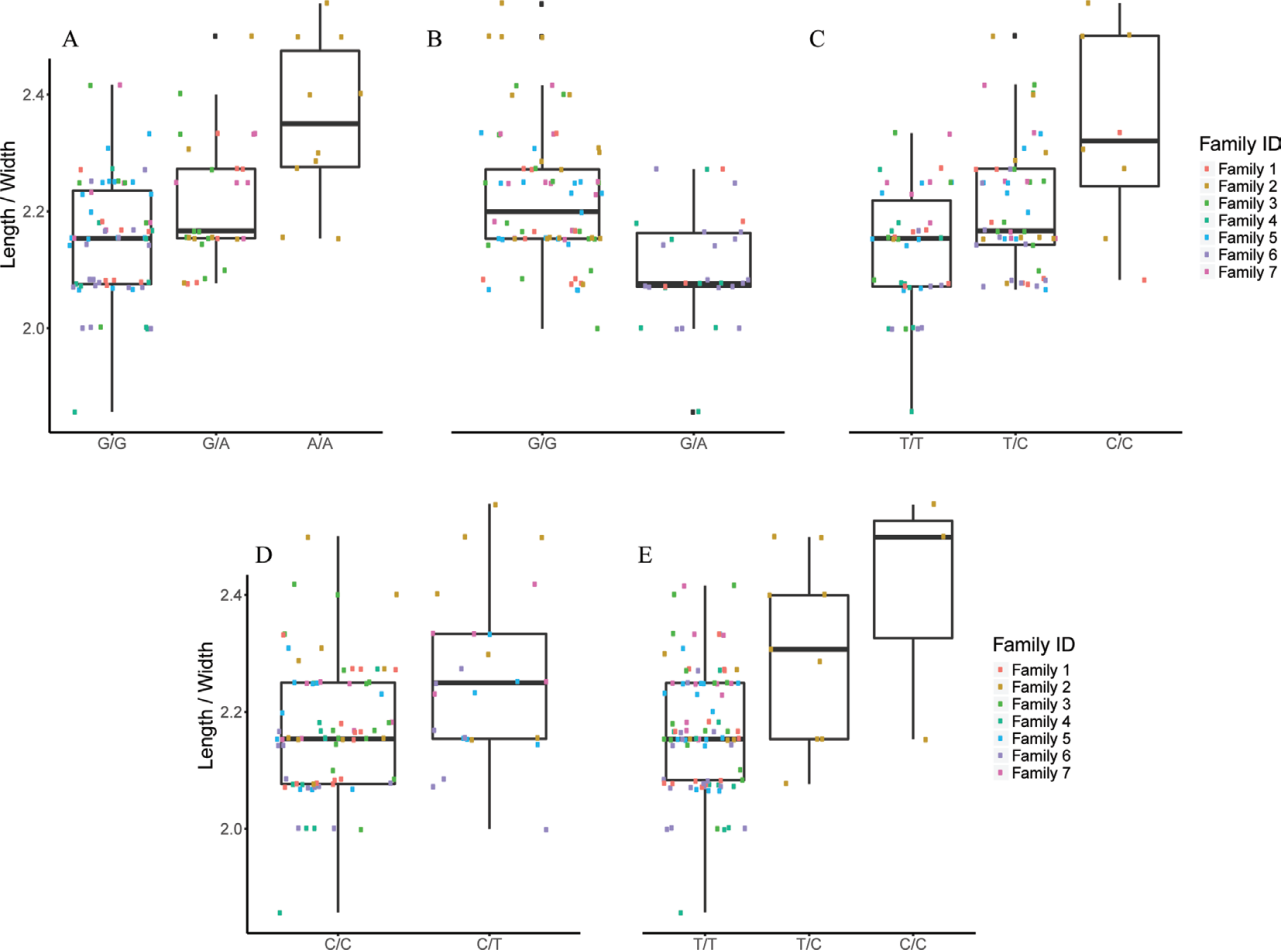
The effect of each of the selected SES SNPs associated with length/width. **(A-E)** Boxplots of selected SNPs. **(A)** chr1:20827142, **(B)** chr3:9671223, **(C)** chr6:23799286, **(D)** chr13:9665394, **(E)** scaffold5661:35982.

Feature selection methods generate groups of SNPs that are associated with a phenotype en masse. Therefore, FS is a valuable family of methods for association analysis. We performed FS with 10 models (8 variants of SES and 2 variants of OMP), and from each model, we extracted the median squared error as an evaluation metric (**Figure 2**). All OMP models were inferior to SES. The best models for Fat and Weight have been constructed by SES algorithm (significance threshold equal to 0.01; number of condition set equal to three). The best model for Tag weight and Length/Width ratio prediction was the model constructed by variables retrieved from SES with size of condition set equal to two. The selected features of the best model, for each phenotype, are presented in **Tables 2**–**5**. SES produced different combination of SNPs (signatures) that have the same predictive strength on each one of the examined traits. In **Tables 2**–**5**, we illustrate one of these combinations, while the rest are illustrated in **Supplementary Tables 2–5**. Finally, the effects of all selected SES SNPs (17 in total, out of which 6 were also found in GWAS) from all traits are presented in **Figures 3**–**6**.

**Table 2:** Selected SNPs from SES algorithm with significance threshold equal to 0.05 (best method based on median squared error score).

Variables	Locus	P-value	Beta coefficient	Threshold	GWAS	Conserved	Position
Fat							
Chr13:1098152	Rho-related GTP-binding -like	0.007	1.60	0.01	–	–	3’ UTR
Chr21:19924408	–	0.006	−1.238	0.01	–	–	–
Chr8: 1385781	Protection of telomeres 1	0.0024	1.55	0.01	–	✓	Intron
Scaffol8147:18634	Death-associated kinase 3-like	0.015	0.7	0.05	–	✓	Intron
Chr7:2453106	Solute carrier family 41 member 1-like isoform X1-2	0.046	0.86	0.05	–	–	Intron
Chr4:23265532	NT-3 growth factor receptor isoform X1	0.017	−2.25	0.05	–	–	Upstream

**Table 3:** Selected SNPs from SES algorithm with significance threshold equal to 0.05 (best method based on median squared error).

Variables	Locus	P-value	Beta coefficient	Threshold	GWAS	Conserved	Position
Weight							
Chr1:16636968	Ethanolamine-phosphate, cytidylyltransferase-like	0.0006	121.84	0.01	✓	✓	3’ UTR
Chr6:12617755	Myosin-7-like isoform X1,short transient receptor potential channel 4-associated	0.0024	138.07	0.01	✓	✓	Upstream
Chr8:11613979	Semaphorin-3A	0.0114	99	0.01	–	✓	Intron
Chr16:2232897	Acetylserotonin O-methyltransferase-like, LBH-like isoform X1	0.0022	−193	0.01	✓	–	3’ UTR
Scaffold29:195838	Mitogen-activated kinase-binding 1-like	0.0285	64.669	0.05	–	–	Intron
Chr24:8282385	STE20-related kinase adapter beta	0.0022	160.80	0.05	–	✓	Downstream
	Trafficking kinesin-binding 2 isoform X1						Upstream

**Table 4:** Selected SNPs from SES algorithm with significance threshold equal to 0.05 (best method based on median squared error score).

Variables	Locus	P-value	Beta coefficient	Threshold	GWAS	Conserved	Position
Tag Weight							
Chr2:2623351	Tetratricopeptide repeat 36	0.0019	4.577	0.01	✓	–	Upstream
Chr13:20883924	DNA repair RAD50	0.0127	2.678	0.01	–	✓	Intron
Chr13:20975921	RNA-binding 27 isoform X1	0.0073	1.810	0.01	✓	–	Intron
Chr22:18343985	Zinc finger BED domain-containing 4-like	0.0117	−1.967	0.01	✓	–	Upstream
	Midasin isoform X2						Downstream
Scaffold4139:36071	Predicted uncharacterized protein LOC106518831, partial	0.033	−0.634	0.01	–	✓	Upstream
Chr15:3260819	Follistatin-related 1-like	0.0124	3.106	0.05	–	–	Downstream
Chr20:6671436	UBA-like domain-containing 1	0.021	2.665	0.05	–	✓	2nd
Chr22:14483563	Exostosin-1-like	0.0448	4.898	0.05	–	–	Intron
Scaffold14083:12192	–	0.042	−1.349	0.05	–	–	-

**Table 5:** Selected SNPs from SES algorithm with significance threshold equal to 0.05 (best method based on median squared error score).

Variables	Locus	P-value	Beta coefficient	Threshold	GWAS	Conserved	Position
Length/Width							
Chr6:23799286	Phosphatase 1 regulatory subunit 3D-like	0.0052	0.0397	0.01	–	✓	3d
Chr1:20827142	Upstream: mucin-5AC-like isoform X1	0.049	0.026	0.01	–	✓	Upstream
Chr13:9665394	ATP-dependent RNA helicase DHX33	0.0211	0.048	0.01	–	✓	3d
Chr3:9671223	A-kinase anchor 9 isoform X3	0.0144	−0.0597	0.01	–	✓	2nd
Scaffold13177:8369	Phosphatase 1 regulatory subunit 3C	0.015	0.057	0.01	–	✓	Downstream
Chr8:11613979	Semaphorin-3A	0.0193	−0.025	0.05	–	✓	Intron
Chr22:2545133	Neurexin-3b isoform X3	0.049	−0.029	0.05	–	–	Intron
Scaffold5661:35982	–	0.049	0.031	0.05	–	✓	–

#### Selected SNPs for Fat Content

The selected variables/SNPs associated with Fat content (%) at harvest, retrieved from SES algorithm (threshold 0.01), recovered three SNPs, out of which two were located within or proximal to an annotated gene (**Table 2**). The first annotated SNP is located within “telomeres 1 (POT1)” gene (chromosome 8), a region found conserved in other species as well (Medaka, Asian swamp, Asian sea bass). The second SNP was located within the “Rho family GTP-binding” gene (chr13:1098152). However, when lowering the significance threshold to 0.05, the number of SNPs increased to six (**Table 2**).

#### Selected SNPs for Weight at Harvest

Four selected variables associated with weight at harvest (800 g average weight at harvest) have been retrieved from SES algorithm with number of condition set equal to three. The first was found in chromosome 1 (chr1:16636968) on “ethanolamine phosphate cytidylyltransferase-like” gene, the second (chr6:12617755) in a conserved region upstream of “myosin-7-like” gene, the third (chr8:11613979) was located in “semaphorin-3A” gene (Conserved in Asian sea bass, Asian swamp eel) and upstream of ‘Piccolo’ gene, and another one (chr16:2232897) and the fourth on two overlapping genes acetylserotonin O-methyltransferase-like and LBH-like isoform X1. When lowering the significance threshold to 0.05, four SNPs were added to the signatures, retrieving two more annotated genes (**Table 3**).

#### Selected SNPs for Weight at Tagging

Five SNPs were associated with Tag Weight, as retrieved from SES algorithm (**Table 4**). The first was found at “RNA-binding 27 isoform X1” gene (chr13:20975921), the second upstream from “Tetratricopeptide repeat 36” gene (Chr2:2623351), the third at “DNA repair RAD50” gene (chr13:20883924), the fourth upstream from “tectonin beta-propeller repeat-containing 2” gene (chr22:18343985), and the fifth (scaffold4139:36071) was not in an annotated region. Lowering the significance threshold to 0.05, four annotated SNPs were added to the discovered signatures (**Table 4**).

#### Selected SNPs for Length/Width Phenotype

Finally, five SNPs were associated with Length/Width ratio (at 750 DPH) as retrieved from SES algorithm (**Table 5**). The first SNP (chr6:23799286) was located on the “phosphatase 1 regulatory subunit 3D-like.” The second SNP (chr16:2232897) was located in two genes “acetylserotonin O-methyltransferase-like” and LBH-like isoform X1. The third SNP (chr13:9665394) was located in “ATP-dependent RNA helicase DHX33,” the next one in “A-kinase anchor 9 isoform X3,” and the last one (scaffold13177:8369) downstream of phosphatase 1 regulatory subunit 3C.

## Discussion

Here, we present a family-based approach for the discovery of genetic variants that are significantly associated with a set of phenotypes with economic importance for the farmed gilthead sea bream. The application of these methods on seven families, each measured on four phenotypes, revealed several genetic signatures that may be used for genomic selection. Various QTL affecting growth, morphology, and stress-related traits have been detected using microsatellite markers in gilthead sea bream (Boulton et al., 2011; Loukovitis et al., 2011; Loukovitis et al., 2012; Loukovitis et al., 2013). Some of those QTL have been verified in genetically unrelated populations (Loukovitis et al., 2016). However, no association study using SNP markers was available for production traits in sea bream except this by Palaiokostas et al. (2016) on pasteurelosis. Our study fills this gap enabling for the first time a genomic scan for SNPs that are linked to important traits. We applied two intrinsically different methods. The first is a typical GWA study that examines variants independently, and the second is a family of methods (SES and OMP) that generates signatures with multiple variants.

The sample size of our study (N = 103) might indeed produce some artifacts of this kind. Nevertheless, the analysis pipeline that we apply (SES) is specially tailored for small or moderate sample sizes in order to detect statistically significant QTLs. We anticipate that a future study with greater sample size will refine our findings and might locate additional important QTLs.

In GWA analysis after the LD-pruning, we found 497 independent SNPs. It expected the LD-pruning to reduce drastically the number of SNPs. Studies has shown that a strict LD filters like the one that we applied has minimal effect on the predictive accuracy of the remaining SNPs (Palaiokostas et al., 2019). In general, we noticed a concordance between the SNPs discovered by GWAS and SES. Both methods include tests for SNP–phenotype statistical association, whereas OMP conducts residual-based tests for SNP association. SES algorithm attempts to identify specific sets of SNPs that model a specific phenotype, whereas the typical GWAS pipeline reveals statistical associations. An interpretation of the significance of the SNPs that were located from GWAS but not from SES is that these SNPs do not have a direct effect. Or else, the effect of these SNPs can be eliminated by conditioning on the SNPs that SES revealed. For example, two SNPs that were identified from the typical GWAS, to be associated with weight at tagging (chr13:20975921, chr13:20975924), were marked by SES as equivalents. SES was built upon MMPC algorithm (Tsamardinos et al., 2003). The difference between these two algorithms is that MMPC does not return multiple solutions. MMPC was shown to achieve excellent false positive rates (Aliferis et al., 2010). Seen from the biological perspective, multiple equivalent signatures may arise from redundant mechanisms, for example, genes performing identical tasks within the cell. For example, Ein-Dor et al. (2005) demonstrated that multiple, equivalent prognostic signatures for breast cancer can be extracted just by analyzing the same dataset with a different partition in training and test set, showing the existence of several loci that are practically interchangeable in terms of predictive power. SES was tested against LASSO (Lagani et al., 2017) with continuous, binary, and survival target variables, resulting in SES outperforming the LASSO algorithm (Groll and Tutz, 2014) both in predictive performance and computational efficiency. Overall, SES seems to be performing well in smaller datasets, while OMP is known to perform better in larger datasets (Tsagris et al., 2018b). A known limitation in every GWA study is that the power to detect small QTL effects is limited by the number of samples. An under-powered GWA study may fail to detect some associations, whereas the detected signals might be inaccurate in terms of location and/or biological interpretation. The sample size of our study (N = 103) might indeed produce some artifacts of this kind. Nevertheless, the analysis pipeline that we applied (SES) is specially tailored for small or moderate sample sizes in order to detect statistically significant QTLs. We anticipate that a future study with greater sample size will refine our findings and might locate additional important QTLs. Our findings highlight novel SNPs found within or close to coding genes that are significantly associated with our focal traits of interest in sea bream. However, multiple of those genes have been linked with such traits in other species as well. Multiple interesting genes were associated with fat content. For example, one SNP locus is linked with the gene Rho-GTP binding, which is involved in adipogenesis in mice, (Sordella et al., 2003). This gene and its regulator (p190-B RhoGAP) seem to have a key role in the outcome of the differentiation of mesenchymal stem cells to either adipocytes or myocytes (Sordella et al., 2003). Another SNP associated with fat was located on neurotrophin-3 (NT-3), a gene with well-recognized effects on peripheral nerve and Schwann cells, promoting axonal regeneration and associated myelination (Yalvac et al., 2018). NT-3 increases muscle fiber diameter in the neurogenic muscle through direct activation of mTOR pathway and that the fiber size increase is more prominent for fast twitch glycolytic fibers. Thus, fat content seems to be influenced greatly by few genes with well-known role in adipogenesis.

Regarding the loci associated with weight and tag weight, we identified 15 genes in total. Interestingly, although those two traits represent the same trait at different stages, we found no gene associated with both. There are many reasons for such result. One reason may be due to the low power of the experiment and the differences in variation in the weight of the fish at different ages. Another reason may be because different genes are affecting growth at different stages of development. A third reason is that may be the gene action is not only additive and epistatic effects exist. In any case, all these scenarios should be further investigated in a more powerful experiment, which would be necessary in any case. The outcome of our analysis revealed SNPs close to very important genes with a well-known role in weight gain–loss, such as Follistatin, myosin-7, and semaphorin (SEMA3A) genes. Follistatin binds and inhibits the activity of several TGF-family members in mice (Lee and McPherron, 2001). Strikingly, follistatin knockout mice have reduced muscle mass at birth underlying the importance of this gene in muscle growth (Lee and McPherron, 2001). Apart from Follistatin, the significant association with Myosin, an actin-based motor molecule with ATPase activity essential for muscle contraction, shows the importance of regulation of muscle growth-related genes in weight. The third gene, semaphorin, is significantly associated with both weight and length/width. SEMA3A gene is involved in synapse development underlying the importance of genes in regulating the nervous system in length. Also, the same SNP, which is located on SEMA3A, was direct upstream of Piccolo gene. Piccolo play roles in regulating the pool of neurotransmitter-filled synaptic vesicles present at synapses. Mice lacking Piccolo are viable; nevertheless, each mutant displays abnormalities. Piccolo mutants reduced postnatal viability and body weight (Mukherjee et al., 2010). Another associated gene, ethanolamine phosphate cytidylyltransferase, plays a role in lipid metabolism and finally EXT1, a gene regulating important developmental pathways such as hedgehog (Siekmann and Brand, 2005).

The compilation of an annotated reference genome for this species has been recently published by the Hellenic Centre for Marine Research (H.C.M.R). (Pauletto et al., 2018) and is also available on the Genome Browser^1^. To our knowledge, this analysis is the first to use this genome as a reference for read alignment and variant calling. Moreover, a literature review did not reveal any study examining the same collection of traits on this species. As an effect, for the moment, we cannot provide a comparative analysis with other studies. Studies on related species include those of Yoshida et al. (2019), which examines weight paper on Nile tilapia, Nguyen et al. (2018), which examines weight on Yellowtail Kingfish, and Yu et al. (2018), which examines weight and total length on Epinephelus coioides. Although our study does not have any common gene with these studies, it is interesting that among these studies, there are also no common genes. This suggests the high genetic variability on these traits across different species and also the need for future studies with higher sample sizes and better coverage that can provide additional insights on the common genetic content of aquacultured species.

## Conclusion

In this study, we employed two different approaches to identify variants associated with growth-related phenotypic traits. Our chosen selected panel combined with the vigorous bioinformatic analyses revealed the most significant SNP loci on the sea bream genome. The discovered candidates are located in the proximity of genes with known involvement in processes related to growth. The combination of these novel loci may lead to the selection of brooders based on specific genetic signatures and can have a great effect on the efficiency of the aquaculture. Moreover, these results could be used to verify or not putative QTL identified in previous studies and could also be used in order to fine map identified QTL in the same population using other types of genetic markers (Chatziplis et al, 2018, in preparation). Following this step, the use of these variants independently as individual SNP (or SNP haplotypes) and/or in combination with other marker information in a MAS program could be a form of direct application in the aquaculture breeding industry. When more dense SNP markers would be available (i.e., SNPchip) for the species and more families from more populations are genotyped (i.e., increase LD), then the application of Genomic Selection will be more feasible and cost effective in terms of any selection accuracy benefits. Nevertheless, our study presents, in a small scale example, the feasibility of GS application as well as the availability of the tools necessary before its application (i.e., GWAS using SNP markers) in an important Mediterranean aquaculture species such as gilthead sea bream.

## Ethics Statement

Animal welfare was achieved according to the “Guidelines for the treatment of animals in behavioural research and teaching” (Guidelines for the treatment of animals in behavioural research and teaching, 1997) (see also Tsakogiannis et al., 2018). All fish utilized in the study were kept in registered and authorized facilities to maintain and perform animal experiments; rearing and sampling followed the guidelines of the Directive 2010/63/EU for the protection of animals used for experimental and other scientific purposes (Official Journal L276/33) (EU, 2010. Directive 2010/63/EU of the European Parliament and the Council of 22 September 2010 on the protection of animals used for scientific purposes. Official Journal of the European Union L 276/33, Animal protection.). In addition, experimental sampling protocols were approved by the IMBBC’s aquaculture department committee and methods were in accordance with relevant guidelines and regulations approved by the Hellenic Ministry of Rural Development and Food and the Regional Directorate of Veterinary Medicine for certified experimental installations (EL 91-BIO-04) and experimental animal breeding (AQUALABS, EL 91-BIO-03). Laboratory personnel include accredited technicians by the Federation for Laboratory Animal Science Associations (FELASA).

## Author Contributions

CT, GP, TM, AK, and DK conceived and designed the study. LP, DC, and CT designed and performed the family selection. AT performed the DNA extraction and ddRAD library preparation. DK performed the bioinformatic analyses with guidance from AK and TM. DK performed the statistical analyses with guidance from MT and IT. DK wrote the first draft of the manuscript. MT, AT, DC, AK, and TM wrote sections of the manuscript. All authors contributed to manuscript revision and read and approved the submitted version.

## Funding

Financial support for this study has been provided by the General Secretariat for Research and Technology (GSRT), Ministry of Education and Religious Affairs, under the National Programme for Competitiveness & Entrepreneurship (EPAN II) funded by National sources and the European Regional Development Fund for the gilthead sea bream. This research was supported in part through computational resources provided by IMBBC (Institute of Marine Biology, Biotechnology, and Aquaculture of the HCMR (Hellenic Centre for Marine Research). Funding for establishing the IMBBC HPC has been received by the MARBIGEN (EU Regpot) project, LifeWatchGreece RI, and the CMBR (Centre for the study and sustainable exploitation of Marine Biological Resources) RI.

## Conflict of Interest Statement

Author LP was employed by company Nireus Aquaculture SA, Greece. The remaining authors declare that the research was conducted in the absence of any commercial or financial relationships that could be construed as a potential conflict of interest.

## Footnotes

1
http://biocluster.her.hcmr.gr/myGenomeBrowser?portalname=Saurata_v1

## Abbreviations

GWAS, Genome Wide Association Study; ddRAD, double digest Random Amplified DNA; SNPs, Single Nucleotide Polymorphisms; FS, Feature Selection; MAS, Marker Assisted Selection; GS, Genomic Selection; QTL, Quantitative trait locus; BIC, Bayesian Information Criterion; LD, Linkage Disequilibrium; QQ plot, Quantile–Quantile plot; SES, Statistically Equivalent Signature; OMP, Orthogonal Matching Pursuit; MB, Markov Blanket; CV, Cross-Validation; PCA, Principal Component Analysis.
