# Supplementary figures and images for: Scanning of Genetic Variants and Genetic Mapping of Phenotypic Traits in Gilthead Sea Bream Through ddRAD Sequencing
Source: Front Genet. 2019 Aug 6;10:675. doi: 10.3389/fgene.2019.00675 (PMC6691846; doi:10.3389/fgene.2019.00675)

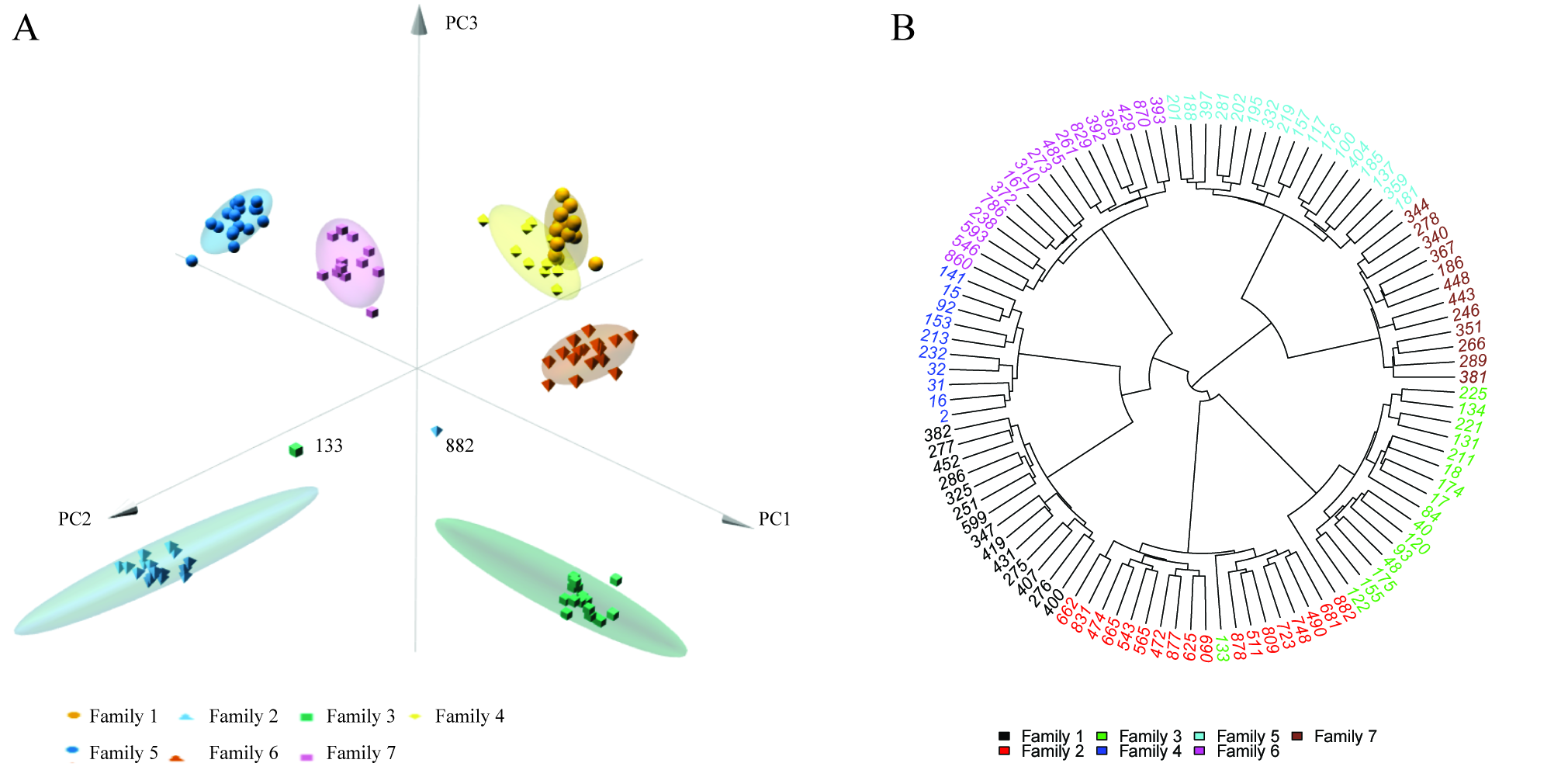

Supplement: Supplementary file 2 [file Image_1.tif]

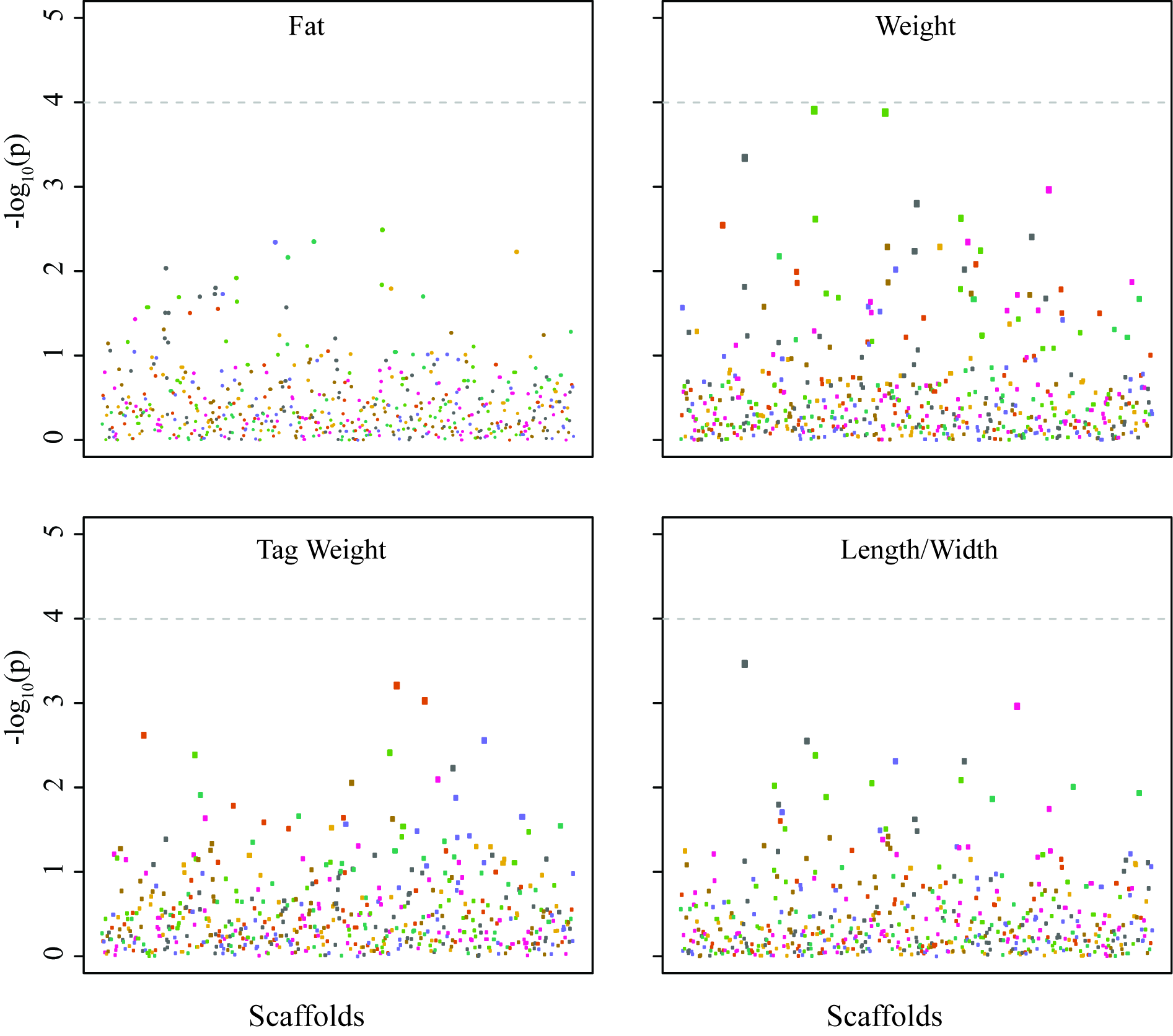

Supplement: Supplementary file 3 [file Image_2.tif]

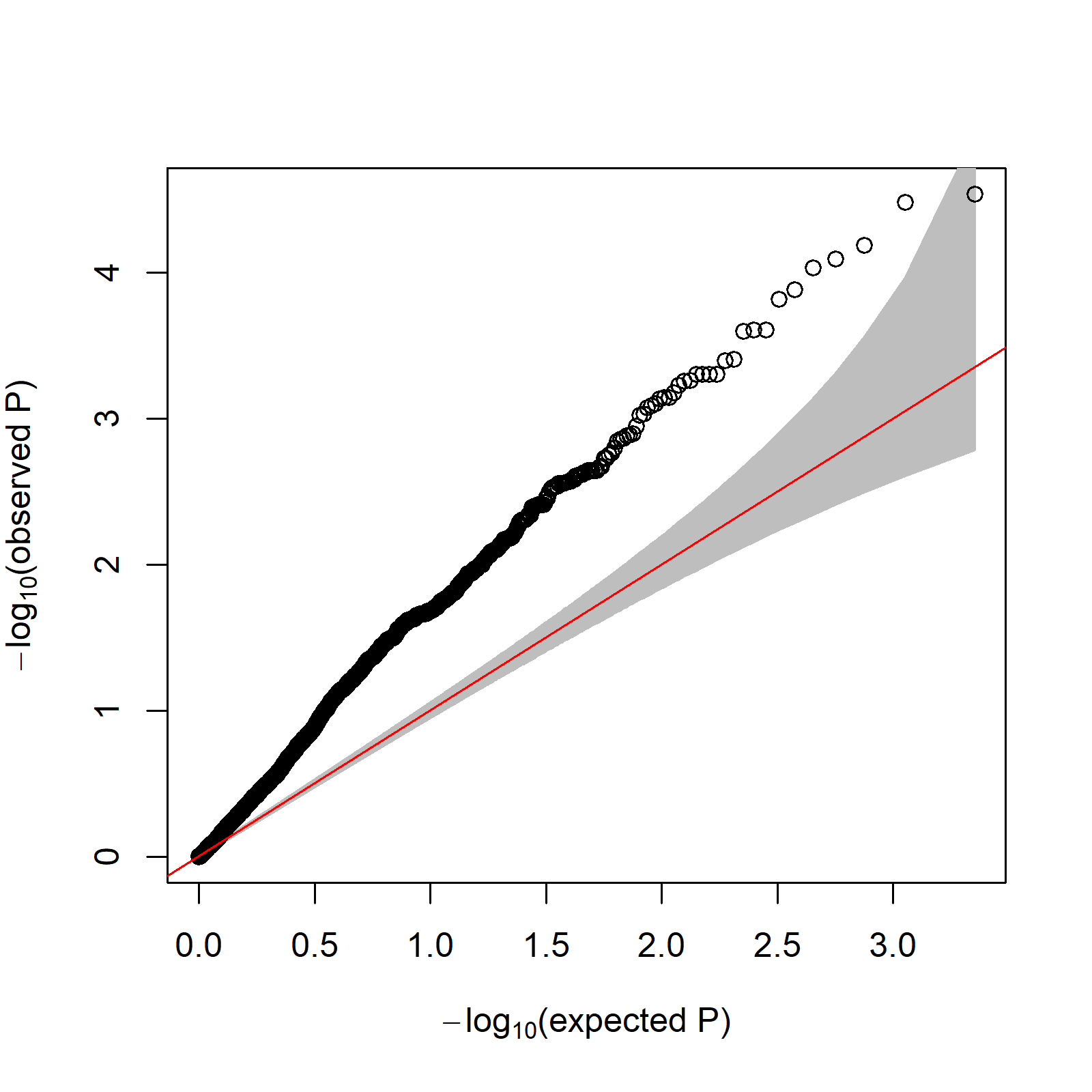

Supplement: Supplementary file 4 [file Image_3.png]

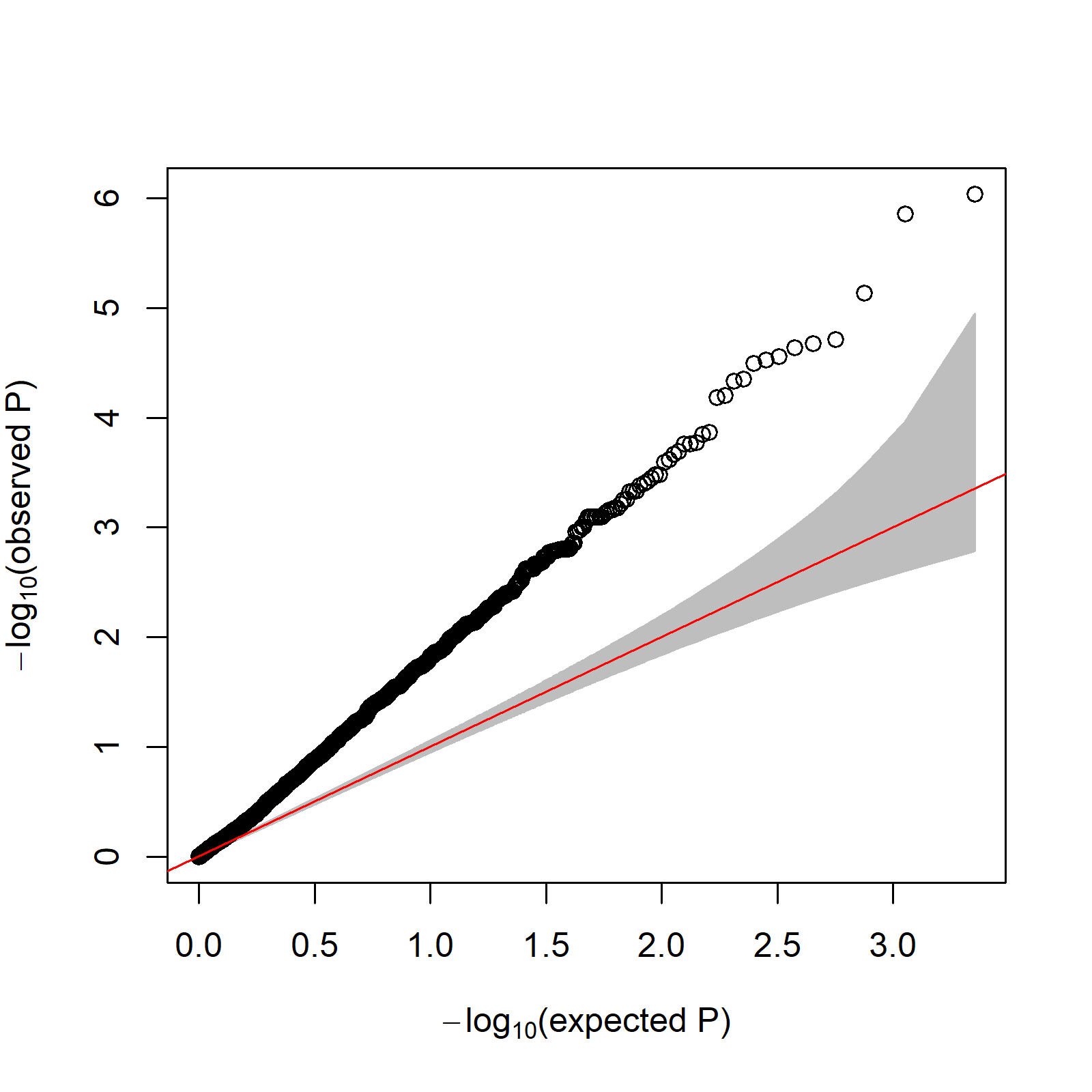

Supplement: Supplementary file 5 [file Image_4.png]

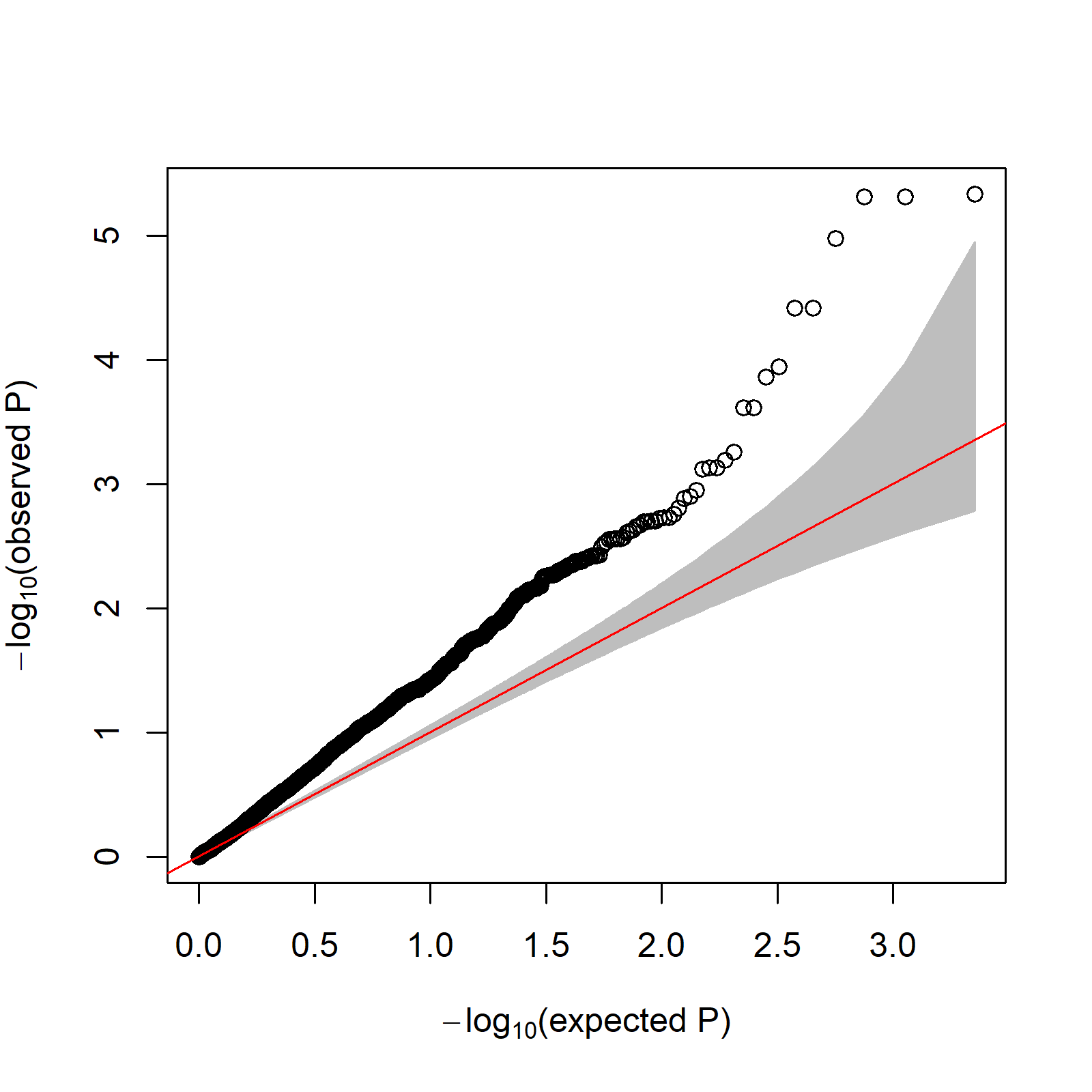

Supplement: Supplementary file 6 [file Image_5.png]

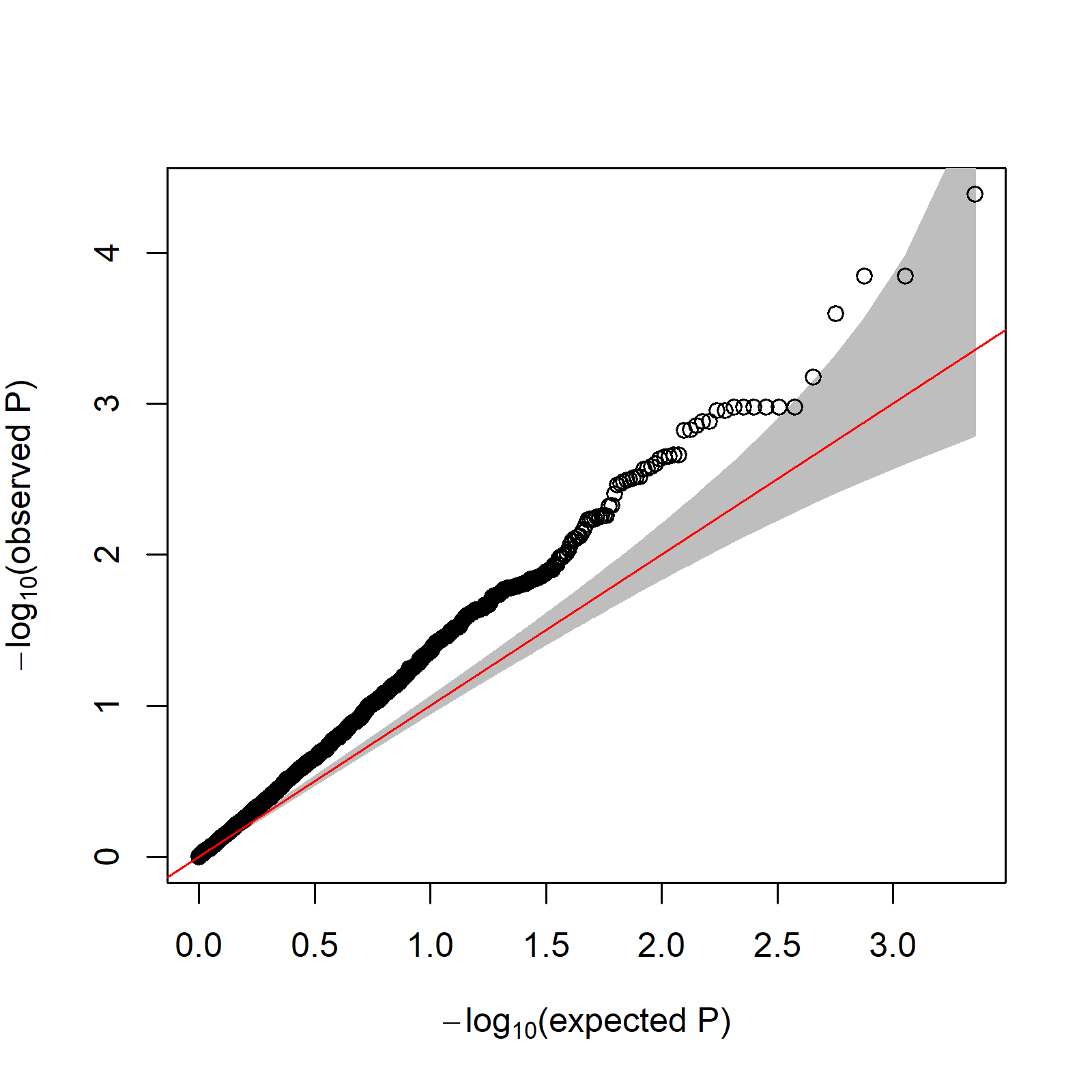

Supplement: Supplementary file 7 [file Image_6.png]
